# Case Illustrative of the Efficacy of the Acetate of Morphine Applied Externally

**Published:** 1826-07

**Authors:** 


### REMARKABLE EFFECT OF ACETATE OF MORPHINE APPLIED EXTERNALLY.

A young woman, 18 years of age, was received into LA PITIÉ, in February, 1826, presenting the following phenomena :—great extenuation—skin hot—pulse small and frequent—tongue pale and dry at the sides—acute pain at the epigastrium and over the whole abdomen, when pressed—cardialgia—nausea—vomiting of every thing, solid or liquid —abdomen tense and swelled—constipation—great sense of debility—painful sensations between the scapulæ—complete loss of voice—urine red and scanty—inquietude—insomnolency.

This young woman had borne a child the year before at Brussels, which was followed by peritonitis that required the most active system of depletion. She had had more than 400 leeches applied to the abdomen and head—and since that period she had never regained her health. Two months previously to the present time, she had had an accession of what was called gastro-enteritis, for which she was treated in one of the MAISONS DE SANTÉ of Paris. In LA PITIÉ M. Serres considered the disease as gastro-entero-peritonitis of the chronic kind, and that it would require some months of the most rigid starvation to remove it. A large cataplasm was ordered to the abdomen, and gum-water to drink. This plan was continued six days, without any benefit. Sugar was the only thing she could keep on her stomach. *15th February*, A blister was applied to the epigastrium ; but this and various other means had no effect in stopping the vomiting. At this time, M. Lambert, a gentleman who has made many experiments on the external application of medicinal substances, proposed the trial of acetate of morphine to the blistered surface. Half a grain was accordingly applied, and, in a few minutes, the vomiting ceased, as if by magic, and the patient passed a better night than she had done for a long time before. The next day the same process was repeated, and the patient slept almost the whole of the day. During the two succeeding days the application failed in preventing the vomiting of substances taken into the stomach, but still maintained its effects of producing sleep. The quantity was gradually augmented to a grain and half. One day it was accidentally omitted, and the patient was thrown into great agitation, and had no sleep that night. But not to detain our readers, the acetate of morphine was still farther increased, and with such good effects that aliment was retained on the stomach, and, when the account closed, the young woman was gaining strength and in a fair way for recovery.

The medical men of the establishment are doubtful whether they had an actual chronic inflammation of the stomach to deal with ; and truly, so are we. It is highly probable that this was one of those gastralgic affections which lead men, especially in France, so much astray, under the false colours of gastritis. Be this as it may, the case may suggest the trial of the medicine above-mentioned, in cases of a similar description. It is proper to observe, that the blistered surface was artificially kept open during the time the morphine was used.—*M. Serres, Archives, Mars.*

